# Effects of motivational interviewing-based adherence therapy for schizophrenia spectrum disorders: a randomized controlled trial

**DOI:** 10.1186/s13063-015-0785-z

**Published:** 2015-06-14

**Authors:** Wai Tong Chien, Jolene HC Mui, Eric FC Cheung, Richard Gray

**Affiliations:** School of Nursing, Faculty of Health and Social Sciences, The Hong Kong Polytechnic University, Hung Hom, Kowloon, Hong Kong, SAR China; Castle Peak Hospital, 15 Tsing Chung Koon Road, Tuen Mun, New Territories, Hong Kong, SAR China; Hamad Medical Corporation, PO Box 3050, Doha, Qatar

**Keywords:** Adherence therapy, Insight into illness, Motivational interviewing, Randomized controlled trial, Schizophrenia spectrum disorders, Symptom severity

## Abstract

**Background:**

Non-adherence to antipsychotic medication is commonly found in schizophrenia and other psychotic disorders, thus forming a major obstacle to long-term maintenance treatment and contributing to high relapse rates. With limited evidence on the success of interventions in enhancing medication adherence, this controlled trial was designed to test and evaluate the effectiveness of an adherence therapy (AT) for outpatients with schizophrenia spectrum disorders, based on a motivational interviewing approach over a six-month follow-up period.

**Methods:**

A single-blind, randomized controlled trial with a repeated-measures, two parallel groups design was conducted in a random sample of 114 participants with schizophrenia spectrum disorders in one community psychiatric nursing service. After pre-test, the participants were randomly assigned to either an eight-session course of AT plus usual care or usual psychiatric care (n = 57 per group). The main outcomes, including medication adherence, symptom severity, insight into treatment, hospitalization rate, and functioning, were measured at baseline and immediately and six months post-intervention.

**Results:**

A total of 110 participants completed this trial and thus the attrition rate was 3.5 %. Results of repeated-measures analysis of variance followed by Helmert’s contrasts test indicated that the AT participants reported significantly greater improvements in their insight into illness and/or treatment, psychosocial functioning, symptom severity, number of re-hospitalizations, and medication adherence (F = 5.01 to 7.45, *P* = 0.007 to 0.030) over six months follow-up, when compared with usual care.

**Conclusions:**

Motivational interviewing-based AT for people with schizophrenia can be effective to reduce symptom severity and re-hospitalizations, and improve medication adherence, functioning, and insight into illness and/or treatment over a medium term (six months) period of follow-up. Further study on the effects of AT in people with psychotic disorders in terms of diverse sociodemographic and illness characteristics, and a longer term (for example, over 12 months) follow-up period is recommended.

**Trial registration:**

The trial was registered at Clinicaltrials.gov (identifier: NCT01780116) on 6 July 2014.

## Background

People with a diagnosis of schizophrenia spectrum disorders, such as schizophrenia, schizophreniform, and schizoaffective disorders, constitute about 60 to 70 % of psychiatric patient populations served by community mental health services [1]. The use of antipsychotic medication in acute, chronic, and maintenance stages of the illness for symptom reduction, improvements of psychosocial functioning, and relapse prevention in these psychotic patients is widely accepted. Nevertheless, systematic reviews on clinical trials have suggested that levels of adherence to oral antipsychotics among these patients were generally poor, ranging from 40 to 70 % [2,3]. Despite the advent of new (atypical) antipsychotics with less side-effects, some adverse effects of these novel drugs, such as tardive dyskinesia and obesity, are highly intolerable and detrimental to patients. While there has been increasing number of patients using these atypical antipsychotics, it appears that little progress has been made on increasing medication adherence in patients with schizophrenia. Continuous efforts are required for the design of innovative and effective interventions, which can be evaluated in improving medication adherence and other patient outcomes among patients with schizophrenia, thus reducing their rate of relapse occurrence [4].

Among various factors such as long duration and serious adverse effects of medication and lack of social and family support [5,6], a lack of insight into the illness and medication taking is one of the most important inhibiting factors influencing medication adherence to antipsychotics and other treatments in patients with schizophrenia spectrum disorders [7]. In addition, acute and severe psychopathology (for example, suspicions and delusional beliefs), and any delay in seeking treatment during the early stages of schizophrenia, can render it difficult for prompt and efficacious interventions and understanding about the significance of medication adherence, thus reducing the likelihood of their adherence behaviors [6,8].

‘Adherence’, sometimes used interchangeably with ‘compliance’ in the literature, means that a patient accepts the advice of healthcare professionals to take medication according to a medical prescription. Indeed, it also reflects the recognized needs for, and importance of taking the prescribed medication from the patient’s perspective [2,9,10]. Systematic reviews on recent clinical trials indicate that current multi-faceted and complex psychosocial interventions for people with schizophrenia can only demonstrate modest and inconsistent effects on improving patients’ treatment adherence and/or other psychosocial outcomes over a short-term follow-up period (for example, six months) [2,3,11]. For instance, psycho-educational programs for people with psychotic disorders, which aimed to enhance knowledge about mental illness and its medications and/or treatments, were found to be non-significant in promoting positive attitudinal and behavioral changes in these patients’ treatment adherence [1,6].

Neither psycho-educational interventions nor behavioral modifications have been found to have a significant effect on medication adherence. One recent controlled trial reported that psycho-educational or behavioral management programs for people experiencing an acute episode of psychosis could significantly reduce their symptom severity, yet no significant effect was found on the patients’ adherence rate, insight to treatments, nor functioning [12]. Researchers are unable to confirm or explain the mechanisms of change induced by strategies in improving medication adherence used, but in order to enhance medication adherence, most recommend that important factors such as patients’ knowledge, attitude, and perceived (or experienced) stigma towards the illness and its treatment, and therapeutic alliance with professionals should be considered [13].

In addition, recent studies have suggested that patients’ adherence to medication could be improved by assisting them in understanding and accepting their illness and its treatment, and in coping with those problems concerning the medication used and its adverse effects [5-7]. Schizophrenia sufferers with a better insight into their illness can demonstrate a better treatment adherence; whereas, a greater perceived susceptibility to re-hospitalization is associated with an increased level of adherence to treatment [3,14]. Motivational interviewing has recently found to be particularly useful for people with addictions or high resistance or reluctance to treatments who are ambivalent to changing their behaviors [11]. This therapeutic approach to behavioral intervention has recently been adopted to enhance adherence to medication in schizophrenia, with positive preliminary evidence on reducing patients’ psychotic symptoms and relapse rates [8,11]. These positive findings suggest a need for further scrutinizing of the effects of this motivational and self-empowered (both cognitive and emotional focused) approach in different patient outcomes in terms of not only symptom severity and relapse from the illness, but also patients’ medication adherence, insight into illness and/or treatment, and psychosocial functioning in those with poor adherence to antipsychotic medication.

Recent clinical practice guidelines, such as the 2014 National Institute of Health and Care Excellence guidelines, recommend an assessment of individual patients’ motivation and potential barriers to adherence to their prescribed medication and treatment regimen prior to any medical treatment or psychosocial intervention for people with schizophrenia [15]. Nevertheless, the most recent systematic reviews on compliance therapy for people with psychotic disorders suggested that existing approaches to medication compliance (that is, merely following what has been prescribed) could not fully support their benefits on patient functioning and relapse prevention, as well as the generalization of their findings across cultures [3,11].

Based on the preliminary evidence on adherence therapy (AT), an innovative model of a 12-session course of AT for people with schizophrenia was developed and modified from Kemp *et al.* [8]. The AT mainly involved a combination of techniques in motivational interviewing, cognitive behavioral therapy, and psycho-education. It was tested in the United Kingdom and Thailand, with positive results on reducing symptom severity and relapse rates, and enhancing levels of medication adherence in schizophrenia [5,15]. Gray *et al.* [11] modified this AT program into a brief (eight session) course based mainly on motivational interviewing, coping, and problem-solving skills. The modified program conducted in a few European countries demonstrated significant improvements in psychotic symptoms and relapse prevention in schizophrenia over a six-month follow-up period, but not in their insight into illness, functioning, and quality of life.

Therefore, this randomized controlled trial was designed to test the effects of a modified AT program for Chinese outpatients with schizophrenia spectrum disorders (together with principles of cognitive behavioral therapy), as recommended by Gray *et al.* [16]. We hypothesized that that the participants who received AT would demonstrate significantly greater improvements in symptom severity at immediately and six months after completion of the intervention, compared with treatment as usual (TAU). In addition, we also hypothesized that the AT participants would show greater improvements in secondary outcomes, including medication adherence rate, re-hospitalization rate, psychosocial functioning, and insight into illness and/or treatment, over the six-month follow-up period.

## Methods

**Fig. 1:**
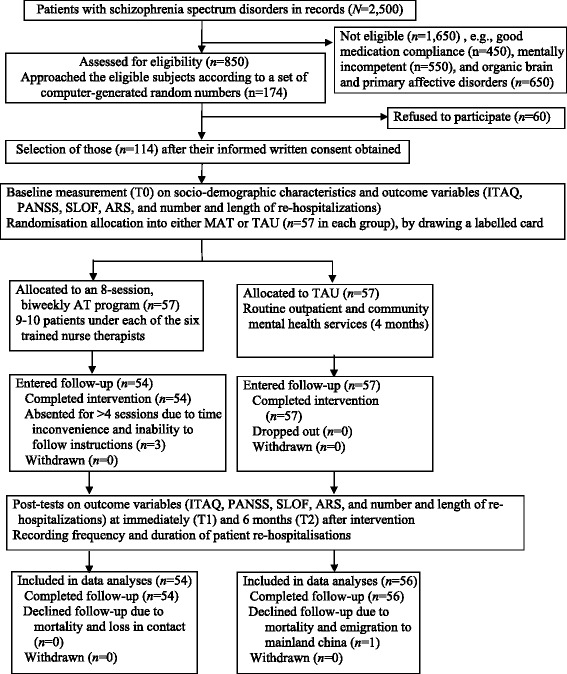
A flow diagram of the procedure of this clinical trial. After confirming eligibility and written consent, 114 patients with schizophrenia spectrum disorders were randomly selected from a patient list and randomly assigned into one of the two study arms (motivational interviewing-based adherence therapy or treatment as usual) after completing the baseline measurements. Fifty-four participants in the adherence therapy and 56 in the treatment-as-usual group completed one to three post-tests over a six-month follow-up period, and finally included in the data analyses. Only four participants dropped out during the study period. AT, Adherence Therapy; CPNS, Community Psychiatric Nursing Service; ITAQ, Insight and Treatment Attitude Questionnaire; PANSS, Positive and Negative Syndrome Scale; SLOF, Specific Level of Functioning Scale; TAU, Treatment as usual

This was a single-blind, randomized controlled trial of a motivational interviewing-based medication AT program for patients with schizophrenia spectrum disorders, using a repeated-measures, parallel groups (AT versus TAU) design. Fig. 1 shows the flow diagram of the trial procedure according to the revised version of the Consolidated Standards of Reporting Trials statement [17]. Ethical approvals of this trial were obtained from the Human Subjects Research Ethics Committee of The Hong Kong Polytechnic University (reference number: HSEARS20120101004), and the community nursing service under study. Written informed consent was obtained from individual participants after clear explanation of the purpose and procedure of the study by the first author.

### Participants and recruitment

The study was conducted between December 2012 and January 2014 at one Community Psychiatric Nursing Service (CPNS) in the New Territories of Hong Kong, which served about 20,000 patients discharged from two regional mental hospitals. There were more than 2,500 potential participants (12.5 % of the total patient population) primarily diagnosed with schizophrenia spectrum disorders such as schizophrenia, schizoaffective disorder, and schizophreniform disorder. A research assistant (RA) checked the patient records in the CPNS and found that 850 patients met the study criteria. These patients were assessed by their attending psychiatrist to confirm their mental competence for participation in the study (that is, being able to understand and complete the study questionnaires). A list of eligible participants was developed in alphabetical order of their surnames. A set of computer-generated random numbers provided by an independent statistician, who was blind to the patient list and not involved in other parts of the study, was used for participant selection from the list of eligible patients. Of 174 eligible participants approached individually by the RA, 114 participants (65.5 %) agreed to participate after providing their written consent.

Participants were included if they were: (a) aged between 18 and 60 years, Hong Kong residents speaking in Mandarin or Cantonese; (b) having a primary diagnosis of schizophrenia spectrum disorders in the past five years, according to the criteria of the revised fourth edition of Diagnostic and Statistical Manual for Mental Disorders (DSM-IV-TR) [18]; and (c) had poor adherence to medication as indicated by a Drug Attitude Inventory score of 11 or below [5], and/or recent history of non-adherence to antipsychotic medication. Non-adherence to medication was defined as a recent history of cessation of oral antipsychotics associated with psychiatric admission or for more than one month at a time [3,11], or missed an average of more than two doses per week in the past three months [3,5] according to the assessment by their attending psychiatrist or community psychiatric nurse and/or self-reported by patients themselves. Exclusion criteria of this study were those patients who had: (a) regular depot or intramuscular injection(s) only; (b) co-morbidities of learning disability, organic brain disease, and/or cognitive impairments; (c) previous participation in any medication management program; and/or (e) hostel (or halfway house) residents supervised by mental health workers to take their medications.

Those patients who were randomly selected (n = 114) and completed the baseline measures (T0) were randomly assigned by the RA into one of the two study arms, namely, AT or TAU, (n = 57 in each group), by drawing a labeled card (1 = AT, 2 = TAU) from an opaque envelope. Those 57 participants in the AT were also assigned to one of six trained community psychiatric nurses according to their sequence of date of home visit. The lists of participants and their intervention assignment were concealed to the researchers, assessors, and CPNS staff until completion of all post-tests.

### Sample size calculation

The sample size calculation was based on the average effects size on symptom severity (Positive and Negative Syndrome Scale; 0.48 to 0.54) and level of medication adherence (Positive and Negative Syndrome Scale; 0.46 to 0.52) at immediately post-intervention in four clinical trials of AT [8,16] or psycho-education programs with medication management as core components in schizophrenia [1,19]. With the level of statistical significance set at *P* = 0.05 (two-sided) and a study power of 80 %, the sample size was 47 per group to detect an average effect size (Cohen’s d) of 0.50 on the above two outcomes, using G*Power 3.1 for Windows [20], taking into account an attrition rate of 20 % [10,19]. Hence, 114 participants were randomly selected (n = 57 in each group).

### Interventions

Motivational interviewing-based AT in this study was based on Gray *et al.*’s [11] eight-session AT using the motivational interviewing technique (and principles of cognitive behavioral therapy). Motivational interviewing techniques involving cognitive, motivational, insight-inducing, and behavioral training are viewed as particularly useful for people with addictions and/or strong resistance and ambivalence to treatment regimen and adherence [5]. Principles of motivational interviewing were adopted to facilitate a non-confrontational approach to the participants, including: (a) expression of empathy; (b) creating discrepancy or challenges with false beliefs or myths; (c) avoiding arguments and anger; (d) rolling with resistance; and (e) supporting self-efficacy and self-empowerment [8]. Whilst it was considerably more difficult to discuss issues in motivation and attitudes towards treatments with participants expressing severe psychotic symptoms (such as delusion, apathy, and anhedonia), motivational interviewing combined with knowledge acquisition and training in problem-solving and coping skills, could be useful for focusing on specific impacts of illness behaviors on these patients, and provide them with opportunities to engage and discuss their ambivalent attitudes towards their illness behaviors, treatments, and possible consequences of non-adherence [11,12].

The AT (a four-month program) consisted of three phases in which eight two-hour sessions were held at participants’ homes every two weeks by one of the six trained community psychiatric nurses. This AT was validated and agreed by two psychiatrists, two community psychiatric nurses, and two outpatients with schizophrenia to ensure feasibility and cultural appropriateness with the local community mental health services; only a few re-arrangements of the topics were made in the third, fourth, and sixth session (perceived barriers to medication adherence; social stigma and family support within a cultural context; and impacts of stigma towards medication on patients’ behaviors and coping, respectively) [21,22]. An outline of the AT program is presented in Table 1. The first phase of AT (two sessions) aimed to engage participants in addressing their needs for, and concerns with medication adherence, facilitating goal and action setting for changes in medication adherence. The second phase (three sessions) focused on education about the mental illness and its treatment, and then explored patients’ strengths and barriers to adherence, assisting them in recognizing social stigma and family support, and developing coping strategies in medication management over months. The third phase (three sessions) aimed to rationalize patients’ beliefs and concerns, manage their perceived or experienced social stigma, and enhance family and social support networks, thus improving relapse prevention and integration into the community.

**Table 1:** An outline of adherence therapy

Phase/Session	Interventions	Main assignments
Phase 1 (2 sessions)	Purposes:	Reviewing antipsychotic medication use and the impacts of psychotic symptoms on medication (and treatment) adherence, the desired and unwanted effects of medication, neuroleptic side effects, and attitude and satisfaction with medication taking.
	(1) To help participants review their past and present states of taking antipsychotics; and	
	(2) To assess knowledge, attitude, and barriers to medication adherence, and plan for problem-solving and improving adherence behaviour using a standard assessment form.	
	Participants identify the present beliefs and concerns, benefits and barriers related to medication and rated the level of distress (1 to 10) attached to each side effect.	Examining and addressing beliefs and concerns towards adherence, and plan for problem-solving.
	Families are asked for their opinions and attitudes on their relative’s attitude towards medication taking.	Homework assignment: Weekly record of adherent behaviour and reasons for adherence or non-adherence.
	Participants are asked to do homework by recording weekly medication adherent behaviors, and both they and nurse therapist keep a record of documentation.	
	The nurse therapist makes an attempt to link medication cessation with relapse. Negative treatment experiences and high level of distress regarding side effects are acknowledged and discussed. Denial of need for treatment is met with gentle enquiry into the ensuring social consequences and lifestyle disruptions.	
Phase 2 (3 sessions)	Purposes:	Revisiting and revising previous goals or add new ones, and their actions.
	(1) To educate about mental illness and the treatment and care required;	
	(2) To review the goals, actions, and adherence records of the last two weeks; and	Recognizing factors that may lead to poor adherence, and developing coping strategies to reduce urges for non-adherence
	(3) To identify barriers to medication adherence and to develop coping strategies, new goals and actions.	Homework assignment:
	Participants’ confusion between symptoms and side effects, and misconceptions of antipsychotic medication is further clarified.	Practicing new actions for maintaining or enhancing adherence. Weekly record of adherent behaviors and reasons for adherence/non-adherence to medication.
	The natural tendency to stop medication whenever the participants feel well is to be discussed, and their meanings attached to medication are explored, that is, an identity as a ‘sick person’.	
	Participants are asked to weigh up the benefits and drawbacks of treatment, and the nurse therapist will focus on the benefits, especially when they emerge spontaneously.	
	Symptoms reported by the participants are fed back as their needs (‘symptoms’) for treatment.	
Phase 3 (3 sessions)	Purposes:	Evaluation of the progress of medication adherence with each participant and his/her change in beliefs/insight into illness and treatment during session 6.
	(1) To rationalize participants’ beliefs and concerns and to prevent relapse;	
	(2) To manage social stigma and enhance social support.	
	Participants are facilitated and encouraged to identify the characteristics of prodromal symptoms and analyze the importance of early intervention to prevent a full-blown episode.	Making future plan with participants to continue self-monitoring of medication adherence and its contractual agreement; and clarifications of means of support from the CPNS, family and services.
	In sessions 7 and 8, the nurse therapist use normalizing rationale to deal with stigma towards the illness and/or medication; suggest an analogy with physical illness requiring maintenance treatment; and highlight illness prevalence with examples of ex-patients who have been successful in coping with similar difficulties as theirs.	Homework assignment:
		Weekly record of medication behaviors and reasons for adherence and non-adherence.
	Participants reframe medication use by participants as a freely chosen strategy to enhance control of quality of life and use metaphors of medication as an ‘insurance policy’ for staying well.	Risk assessment for relapse prevention and a list of risk factors identified and recorded on a standard form.
	A future plan and contractual agreement are made to continue monitoring of medication adherence and means of support from the CPNS, family, and other mental health care services are clarified.	

The AT was carried out by six trained community psychiatric nurses during their home visit for the assigned participants; each of them conducted this therapy for between nine and 10 participants who were randomly selected from between 40 and 45 patients in their caseloads. All participants were asked not to tell their psychiatrists and other staff in CPNS about their participation in the study. While only these six community nurses implemented AT to the assigned patients, other community nurses in the CPNS were concealed to the participant assignments to the interventions. These nurse therapists had received two full-days training by the research team based on both the AT instructor program developed by Gray *et al.* [11] and a psycho-education program for Chinese patients with schizophrenia [1,21]. The trained nurses were provided with supervised practices on AT for at least three outpatients with schizophrenia until the supervisors (the first and second authors) agreed and confirmed the consistency and competency of their implementation of the treatment protocol (over 90 % of items rated as ‘Fully competent’), using a validated competency scale of AT [16]. During the therapy, three sessions were randomly selected for each nurse therapist, audio-taped (with the nurses’ and participants’ prior consents) and assessed by two raters (researchers) to monitor the treatment fidelity using the same competency scale. The fidelity of treatment implementation (rated as ‘Fully competent’) ranged between 90 % and 98 % on the items of the scale (median = 94 %).

The participants in the TAU group received routine community mental health services offered by the CPNS under study and its affiliated outpatient clinic. TAU consisted of psychiatric consultations at the outpatient clinic, home visits by a community psychiatric nurse every four to six weeks with mental health assessments, a brief education on mental illness and its treatments, and referrals to mental healthcare services by a psychiatrist and to social welfare services by a medical social worker.

### Measures

Participants were asked to complete four outcome measures at recruitment (T0) and immediately (T1) and six months (T2) post-intervention by the RA who was blind to their intervention assignment. The primary outcome was symptom severity measured with the Positive and Negative Syndrome Scale (PANSS). The secondary outcome measures included the Insight and Treatment Attitude Questionnaire (ITAQ), Adherence Rating Scale (ARS), frequency and length (days) of psychiatric re-hospitalizations, and Specific Level of Functioning Scale (SLOF).

### Positive and Negative Syndrome Scale

The 30-item PANSS developed by Kay *et al.* [23] assessed the severity of psychotic symptoms in terms of three subscales, including positive symptoms (hallucinations, delusional beliefs, and thought disorganization), negative symptoms (blunted affect, social withdrawal, and lack of spontaneity), and general psychopathology (mannerisms, abnormal posture, and unusual thought contents). Its items were scored on an eight-point Likert scale (from 1-‘Absent’ to 7-‘Extremely’). The scale demonstrated good concurrent validity with the Brief Psychiatric Rating Scale (Pearson’s r = 0.85 to 0.90), test-retest reliability (intra-class correlation = 0.85 to 0.90) and internal consistency (Cronbach’s α = 0.88 to 0.91) in people with psychotic disorders [24].

### Adherence Rating Scale

The ARS developed by Staring *et al.* [13], which was user-friendly and valid and overcame the constraints and difficulties in pill counts, urine tests, and invasive and expensive serum assays [8], measured the level of medication adherence in this study. It was a single-item rated on a five-point scale (1-‘Total non-adherence’, 2-‘Poor adherence’, 3-‘Inadequate adherence’, 4-‘Fair adherence, and 5-‘Good adherence’) by the RA and participants attending a psychiatrist or outpatient clinic nurse (who were blind to their intervention assignment) independently, by asking patients and/or their family members about their drug-taking behaviors and conducting an examination of the patient’s outpatient and CPNS records. Excellent inter-rater reliability (95 to 100 % agreement) and content validity were demonstrated in Coldhan *et al.* [25], a study in American patients with psychotic disorders. If any difference was found on the ratings between the two raters, discussion and final consensus was made by the two raters, with consultation to the patients’ case managers in the CPNS.

### Insight and Treatment Attitude Questionnaire

The 11-item ITAQ assessed patients’ insight into their mental illness and needs for treatments [26]. Items were rated on a 3-point Likert scale (0-‘No insight’; 1-‘Partial insight’, and 2-‘Good insight’); the higher the total score, the better the patient’s insight into the illness and its treatments. The Chinese version used in this study indicated satisfactory internal consistency (Cronbach’s α = 0.82), inter-rater reliability (intra-class correlation = 0.82), and concurrent validity with symptom severity and psychopathology measures (Pearson’s r = 0.56 and 0.60, respectively, both *P* values = 0.001) in Chinese patients with schizophrenia [21,27].

### Specific level of functioning scale

The 43-item SLOF assessed three functional domains of patients with schizophrenia, including self-maintenance and self-care (12 items), social functioning (14 items), and community living skills (17 items) [28]. Its items were rated on a five-point Likert scale (from 1-‘Totally dependent’ to 5-‘Highly self-sufficient’). The Chinese version used in this study demonstrated satisfactory content validity, test-retest reliability (intra-class correlation = 0.80), and internal consistency (Cronbach’s α = 0.88 to 0.96) in Chinese patients with psychotic disorders [29,30].

### Re-hospitalization rate

Number and length (days) of psychiatric hospitalizations over the past four months were reported by participants at pretest and two post-tests, and verified with their clinical records in the CPNS. In addition, the nature of each admission (voluntary or compulsory) was recorded to identify patients’ willingness and involvement in psychiatric treatments.

### Procedure

During home visits, participants completed all of the above-mentioned outcome measures at recruitment (T0) and immediately (T1) and six months (T2) after their completion of the interventions. Participants also complete a sociodemographic data sheet at recruitment, which included patient’s gender, age, education level, monthly household income, and duration of mental illness. Their re-hospitalizations and duration of illness were examined and confirmed with the CPNS records (that is, the electronic patient records system).

### Data analysis

All quantitative data in the AT and TAU group were numerically coded and analyzed on an intention-to-treat basis, using SPSS for Windows, version 20.0 (IBM Corporation, New York, USA). Goodness of fit chi-square test (for categorical data) and independent sample *t* test (for interval or ratio data) were adopted to test the heterogeneity of two groups in terms of their sociodemographic characteristics and mean values of the outcome measures at baseline. Data on AT participants’ attendance, dropouts, receiving other psychosocial interventions, and types and doses of psychotropic medications used over the study period were analyzed. Repeated-measures analysis of variance (ANOVA) test was performed for each of the outcome variables (ITAQ, PANSS, SLOF, ARS, and number and length of re-hospitalizations) to determine the treatment effects in terms of within-group, between-groups, and interactive group-by-time modes [31]. Very few missing data were found and thus were replaced by their group mean values [32]. For those outcomes with significant results of repeated-measures ANOVA test, Helmert’s contrasts tests were performed to test any significant differences on their mean values between groups at each of the two post-tests. The level of statistical significance was set at 5 %.

## Results

### Sample characteristics

A total of 114 participants completed the questionnaires at baseline. Of them, 110 were included in the final data analysis (attrition rate of 3.5 %); three failed to attend more than four AT sessions and one in the TAU group was lost to follow-up. Average attendance to AT sessions was 6.9 (SD = 1.0; median = 6.0, range: 3 to 8).

Sociodemographic (and clinical) characteristics of the 114 participants are summarized in Table 2. About half of them were male (50.9 % and 52.6 % in AT and TAU, respectively), two thirds were diagnosed as schizophrenic (61.4 % and 63.2 %, respectively), and two thirds were recently employed in full- or part-time work (both 63.2 %). The participants had a mean age of 28 to 29 years, mainly ranging from 18 to 49 years (>94 %). Most of them had obtained secondary school or university education (>85 %) and had less than a two-year duration of mental illness (84.2 % and 90.7 %, respectively). All were receiving psychiatric outpatient and/or daytime hospital services. About two thirds were living with their families (66.7 % and 64.9 %, respectively) and 70 to 80 % in independent private or public housing (80.7 % and 75.4 %, respectively). There were no significant differences on all of these sociodemographic characteristics between the AT and TAU group, using the chi-square test (*P* ≧0.12).

**Table 2:** Demographic and clinical characteristics of participants at baseline (N = 114)

Characteristics		AT (n = 57)	TAU (n = 57)	χ ^*2*^, *P*
		f (%)	f (%)	
Gender	Male	29 (50.88)	30 (52.63)	1.30, 0.23
	Female	28 (49.12)	27 (47.37)	
Age (Mean, SD)		(29.21, 9.64)	(28.13, 8.96)	
	18 - 29	18 (31.58)	17 (29.82)	1.68, 0.14
	30 - 39	27 (47.37)	25 (43.86)	
	40 - 49	9 (15.79)	12 (21.05)	
	50 or above	3 (5.26)	3 (5.26)	
Diagnosis	Schizophrenia	35 (61.40)	36 (63.16)	1.08, 0.28
	Other psychotic disorders	22 (38.60)	21 (36.84)	
Employment status	Employed (full-time)	26 (45.61)	25 (43.86)	1.29, 0.21
	Employed (part-time)	10 (17.54)	11 (19.30)	
	Unemployed	14 (24.55)	16 (28.07)	
	Others (for example, an intermittent job)	5 (7.5)	8 (11.9)	
Education level	Primary school	8 (14.04)	9 (15.79)	1.53, 0.12
	Secondary school	41 (71.93)	40 (70.17)	
	University/college	8 (14.04)	8 (14.04)	
Duration of illness (months) (Mean, SD)		(19.91, 11.88)	(20.42, 10.38)	
	<6	20 (35.09)	19 (33.33)	1.33, 0.20
	6 - 12	18 (31.58)	18 (31.58)	
	13 - 24	9 (15.79)	9 (15.79)	
	25 - 36	8 (11.9)	11 (19.30)	
Treatment setting	Outpatient department	57 (100.00)	57 (100.00)	1.41, 0.19
(other than CPNS)	Day hospital/center	9 (15.79)	8 (14.04)	
	Others	10 (17.54)	12 (21.05)	
Living situation	Supervised care	12 (21.11)	11 (19.30)	1.01, 0.31
	Family residence	38 (66.67)	37 (64.91)	
	Living alone	7 (12.28)	9 (15.79)	
Accommodation	Private household	21 (36.84)	20 (35.09)	1.83, 0.10
	Public housing	25 (43.86)	23 (40.35)	
	Others (for example, hostel or long-stay care homes)	11 (19.30)	14 (24.56)	

AT: Motivational-interviewing-based Adherence Therapy, TAU: Treatment as usual

### Results of outcome measures at baseline

The results of all outcome measures (ITAQ, PANSS, SLOF, ARS, and re-hospitalization rates) at baseline are summarized in Table 3. There were no significant differences on their mean scores between the AT and TAU (*P* ≧0.10). The majority (87.9 % in AT and 85.9 % in TAU) were deemed totally non-adherent or poorly to inadequately adherent to medication in both the AT and TAU groups (mean score of 1.48 and 1.39; SD = 0.98 and 1.01, respectively) at baseline. The average number of re-hospitalizations over the past four months were 1.41 (SD = 0.98) and 1.50 (SD = 0.92) in the AT and TAU group, respectively; in which about 65 % were ‘compulsory’ admissions (67 % in AT and 63 % in TAU).

**Table 3:** Results of outcome measures at baseline (N = 114)

Outcome measures	AT		TAU		Unpaired *t* test	*P*
	(n = 57)		(n = 57)			
	M	SD	M	SD		
ITAQ (0–22)^a^	9.12	6.14	9.33	3.31	1.52	0.20
PANSS						
Total score (30–210)^a^	80.19	11.10	81.13	12.01	1.71	0.18
Positive symptoms (7–49)^a^	15.39	5.12	15.11	5.01		
Negative symptoms (7–49)^a^	16.31	5.87	16.45	6.87		
General psychopathology (16–112)^a^	48.49	9.12	49.57	9.88		
SLOF						
Total score (43–215)^a^	140.01	18.22	138.34	17.18	1.49	0.20
Self-maintenance(12–60)^a^	42.11	5.52	40.18	7.08		
Social functioning (14–70)^a^	42.00	5.88	43.01	6.91		
Community living skills (17–85)^a^	55.90	8.01	55.15	8.33		
Adherence rating scale (1–5)^a^	1.48	0.98	1.39	1.01	1.92	0.12
Total non-adherence (f, %)	(22, 38.80)	(20, 35.09)	χ^2^ = 1.48	0.13		
Poor to inadequate adherence (f, %)	(28, 49.12)	(29, 50.88)				
Fair to good adherence (f, %)	(7, 12.28)	(8, 14.04)				
Re-hospitalization						
Number^b^	1.41	0.98	1.50	0.92	1.83	0.12
Duration^c^	9.12	3.98	10.01	4.02	2.33	0.10
DAI (0–22)^a^	8.82	3.03	9.01	3.71	1.51	0.13

AT: Adherence therapy, DAI: Drug Attitude Inventory, ITAQ: Insight and Treatment Attitude Questionnaire, PANSS: Positive and Negative Syndrome Scale, SLOF: Specific Levels of Functioning Scale, TAU: Treatment as usual

^a^Possible range of score in each measure in the parentheses. ^b^Average number of readmissions to a psychiatric hospital or inpatient unit in the past four months. ^c^Duration of psychiatric readmissions at each time point in terms of average number of days of hospital stay over the past four months

### Treatment effects of adherence therapy

The mean scores (and SD) of the outcome measures at pre-test and post-tests in both AT and TAU are shown in Table 4. The results of repeated-measures ANOVA tests indicated the AT group had significant greater improvements over time (group × time interactions) than the TAU group on the participants’ ITAQ, PANSS, SLOF, ARS, and average number of re-hospitalizations. As indicated in Table 4, the interaction (group × time) effects included: reductions in the AT participants’ symptom severities (PANSS score, *F*(1,109) = 7.32, *P =* 0.008; positive symptoms score, *F*(1,109) = 7.28, *P* = 0.008; negative symptoms score, *F*(1,109) = 7.81, *P* = 0.006), with large effect sizes of 0.70 to 0.75; reductions in average number of re-hospitalizations (*F*(1,109) = 5.01, *P =* 0.03), with a moderate effect size of 0.48; improvements in their insight into illness and/or treatment (ITAQ score, *F*(1,109) = 6.58, *P =* 0.021), with a moderate effect size of 0.51; and improvements in functioning (SLOF score, *F*(1,109) = 6.89, *P =* 0.014), with a large effect size of 0.68. In addition, the medication adherence of the AT group showed a significantly greater improvement over time (ARS score, *F*(1,109) = 7.45, *P =* 0.007), with a large effect size of 0.72, when compared with the TAU group.

**Table 4:** Results of repeated-measures ANOVA (group × time) tests for outcome measures at pre- and post-tests (N = 110)

	AT (n = 54)						TAU (n = 56)							
	T0		T1		T2		T0		T1		T2			Effect size
Instrument	Mean	SD	Mean	SD	Mean	SD	Mean	SD	Mean	SD	Mean	SD	*F* ^c^, *P*	
ITAQ	9.12	6.14	11.13	6.89	13.88	6.80	9.33	3.31	9.89	5.81	9.79	6.21	6.58, 0.021	0.51
PANSS	80.19	11.10	74.01	15.10	68.12	14.81	81.13	12.01	81.18	15.23	83.45	14.13	7.32, 0.008	0.71
Positive Symptoms	18.02	4.89	16.13	4.54	14.65	3.98	18.21	4.12	18.23	4.51	18.98	5.86	7.28, 0.008	0.70
Negative Symptoms	20.68	5.01	18.34	5.62	16.71	5.81	20.82	5.76	20.96	5.98	21.38	6.12	7.81, 0.006	0.75
SLOF	140.01	18.22	150.80	22.38	168.12	28.10	138.34	17.18	138.11	20.88	145.12	34.20	6.89, 0.014	0.68
ARS	1.48	0.98	2.12	1.08	3.08	1.24	1.39	1.01	1.45	1.00	1.48	1.01	7.45, 0.007	0.72
Re-hospitalization														
Number^a^	1.41	0.98	1.31	0.92	1.18	1.10	1.50	0.92	1.38	1.09	1.51	0.99	5.01, 0.030	0.48
Duration^b^	9.12	2.98	8.81	5.11	8.18	4.02	10.01	4.02	10.05	8.11	9.01	8.85	3.68, 0.090	0.26

AT: Adherence Therapy, ARS: Adherence Rating Scale; possible score range 1 to 5, with higher scores indicating better adherence to medication, ITAQ: Insight and Treatment Attitudes Questionnaire; possible score range from 0 to 22, with higher scores indicating better insight, PANSS: Positive and Negative Syndrome Scale; possible score range from 30 to 210, with higher scores indicating greater severity of symptoms; the possible scores of Positive and Negative Symptoms subscales range from 7 to 49, SLOF: Specific Levels of Functioning scale; possible score range from 43 to 215, with higher scores indicating higher level of psychosocial and self-care functioning, TAU: Treatment as usual, T0: Baseline measure at recruitment, T1: First post-test at immediately after completion of the interventions, T2: Second post-test at six months after interventions

^a^ Average number of readmissions to a psychiatric hospital or inpatient unit over the past four months, ^b^ Duration of readmissions to a psychiatric hospital or inpatient unit in terms of average number of days of hospital stay over four months. ^c^ *F* values (group × time) for repeated-measures ANOVA test, *df* = 1,109

Results of Helmert’s contrasts test indicated that the AT participants had significantly greater improvements than those in the TAU group on the following patient outcomes at post-tests:

1. Insight into illness and/or treatment significantly increased at both T1 and T2 (mean difference = 1.24 and 4.09, standard error [SE] = 1.10 and 0.60, respectively);
2. Symptom severity (PANSS overall scale, Positive Symptoms and Negative Symptoms) significantly decreased at both T1 (mean difference = 7.17, 2.1, and 4.33, SE = 0.23, 0.05, and 0.92, respectively) and T2 (mean difference = 15.33, 2.62, and 4.67, SE = 0.61, 0.30, and 0.0.31, respectively);
3. Level of functioning significantly increased at both T1 and T2 (mean difference = 12.69 and 23.00, SE = 2.00 and 5.88, respectively);
4. Medication adherence rate significantly increased at both T1 and T2 (mean difference = 0.55 and 1.60, SE = 0.09 and 0.22, respectively); and
5. Number of re-hospitalizations significantly reduced at T2 only (mean difference = 0.33, SE = 0.11); whereas in TAU, it slightly increased across post-tests (from 1.50 at T0 to 1.51 at T2).

Nevertheless, the results also showed that the types and doses of psychotropic medication, nature of admission (voluntary or compulsory) and types and frequency of participation in other psychosocial interventions did not differ significantly between the two groups from T0 to T2 (using repeated-measures ANOVA or Kruskal Wallis test, *P* >0.25). In addition, there were not any significant differences on all mean outcome scores between six AT subgroups in terms of the nurse therapists at pre- and post-tests (using Kruskal Wallis test, *P* >0.10).

## Discussion

This was one of very few clinical trials of AT based on motivational interviewing and cognitive behavioral techniques that was currently available, and found to be successful (effective) in improving adherence to antipsychotic medication among patients with schizophrenia and its spectrum disorders. The results supported the study hypotheses that the participants who received AT could demonstrate significantly greater improvements over a six-month follow-up period in their primary outcome (mental state (symptom severity)) and other secondary outcomes, including insight into illness, functioning, and number of re-hospitalizations, as well as their adherence to medication. In addition, large effects of AT (effect sizes of 0.68 to 0.72) were demonstrated on improving a wide variety of patient outcomes over the medium term (six months) duration of follow-up.

The success in this AT is particularly important to these group of psychotic patients who often have a poor adherence to treatment and a high relapse rate (50 to 70 %) within the first five years of the illness [33], thus having high risks of its recurrence, a long course, and poor prognosis [9]. It is also noteworthy that this motivational interviewing-based AT can improve not only the positive symptoms (such as hallucinations and delusions) that can often be reduced effectively by good adherence to medication [13,16,34], but also those treatment-resistive negative symptoms, for example, amotivation, anhedonia, and social withdrawal. This result might be due to the effects of the motivational interviewing technique, in which the participants were facilitated to non-judgmentally and freely explore and resolve ambivalence on their attitudes and behaviors regarding the illness, its treatment, and related life situations, and to engage them with an enhanced intrinsic motivation to change their treatment- and health-related behaviors, such as medication adherence, self-care, and help-seeking [35]. Other strategies in motivational interviewing, such as showing genuine empathy, acceptance, and envisioning for a better future, could also be helpful in energizing and fostering better initiatives, therapeutic growth, and changes in negative symptoms and passivity in this patient population [13,16].

These findings provide evidence that this AT, modified from Kemp *et al.* and Gray *et al.* in Western culture [8,11], can be a promising intervention across cultures for enhancing positive attitudes and adherence towards antipsychotic medication, and in reducing psychotic symptoms in Chinese people with schizophrenia spectrum disorders. While recent controlled trials on AT showed inconsistent or non-significant results in European countries [5,8,12], the AT used in this study showed significant effects on a variety of patient outcomes, including not only positive symptoms and number of re-hospitalizations, but also those variables often found non-significant in other studies, such as negative symptoms, functioning, and insight into illness and/or treatment [12]. With much more positive findings than other studies of medication adherence or management programs, AT has already overcome several limitations and revealed a few key elements of an effective psychosocial intervention. First, one frequently reported limitation was the difficulty in recruiting non-adherent patients, where the researchers concluded that the insignificant results were, in part, due to the ceiling effect on levels of adherence at baseline provided by those with already good adherence to medication [12,16]. Noting such an effect, participants in this study were only included if they were deemed non-adherent or poorly adherent (ARS mean score of about 1.5 out of 4.0) before the interventions.

Second, several studies reported an oversight of the impact of clinicians’ characteristics and relationship with patients in the effectiveness of psychosocial interventions, recommending that further studies utilize patient-centered healthcare workers that have already been part of the patient’s clinical team [9,16]. The AT was administered by the participants’ attending community psychiatric nurse during home visits. This AT also emphasized participant involvement during the later phases of the intervention, enhancing the sense and feelings of control and self-efficacy in self-regulation and management of the illness and its treatment (and medication), as suggested in other studies as predictive factors of effective psychosocial interventions for people with schizophrenia [22,36].

This study was also one of very few controlled trials to date of AT conducted in Chinese or Asian populations. When compared with other ATs or other interventional studies in Western countries, much higher treatment adherence and lower attrition rates were found in this study, similar to several studies involving Chinese patients [19,21,27]. Such increased adherence and/or retention rates of patients with schizophrenia and other severe mental illnesses may be related to culturally defined perceptions of a strong relationship between patients themselves and their healthcare providers and much better patient compliance with study or treatment protocols [37].

While there are arguments on whether patients’ enhanced insight into illness and/or treatment and adherence behaviors are mediating factors rather than outcome measures of AT [38,39], the AT tested herein can produce positive effects on not only patients’ attitude and behavior regarding medication adherence, but also their mental state and functioning. These positive results that AT can improve the cognitive, psychological, and behavioral domains of its participants, may provide a new understanding and/or direction for further research about the complex relationships between a motivation-enhancing and insight-inducing psychosocial intervention and the above psychosocial and behavioral variables, as well as side effects of medications used and patients’ symptom severity [38].

The AT used in this trial was conducted by six trained community psychiatric nurses. In contrast with recent interventional studies in which there were limited measures in monitoring the treatment fidelity, the AT was guided by a validated manual and delivered by these six nurse therapists with adequate training and supervised practice before conducting the intervention. In addition, a high level of competency in their implementation of the intervention (the median rating on the trained nurses’ competency by two independent assessors was 94 %) was demonstrated. Such a high level of treatment fidelity can increase the treatment integrity, thus enhancing the internal validity of the study [16,27] and the completion rate of this AT program.

This approach to AT showed very clear benefits in terms of psychopathology and treatment insight and adherence for patients with acute to three-year duration of schizophrenia, who indicated poor medication adherence and moderate psychotic symptoms and functioning. The sample recruited could represent those patients with the particular need for an effective medication and illness management during the early stages of psychotic disorders; importantly, it was in contrast to recent AT studies in which most participants were in low to moderate levels of non-adherence to medication [9,13,16]. The participants’ non-adherence rate (76 to 79 %) at baseline measurement in this trial were comparable with most epidemiological and clinical studies for early stages of psychosis [25,39], thus enhancing the generalizability of these findings to current clinical and patient situations.

Recent reviews have suggested that the effects of AT could not be considered substantive in a medium term (six months) follow-up period; similar AT programs with a course of four to six sessions were unable to show significant effects on important patient outcomes, such as positive and negative symptoms and even relapse rate [5,36]. Nevertheless, the results of this controlled trial can confirm the medium-term effect of AT on most of the outcome measures, with an observable reduced frequency of re-hospitalizations over the six-month follow-up period. Previous research has suggested that the effects of AT based on the motivational interviewing technique appeared to be modest and inconclusive [8,12,40]; however, the findings of this trial support and reinforce the importance of such approach and show that it can be effective in improving the psychosocial health outcomes of people in the early stage (less than five years) of schizophrenia spectrum disorders. The findings also echoed the recommendations by Gray *et al.* [16]: that adherence modifying factors essentially centered on improved self-determination, patient choice, and shared decision-making.

Furthermore, AT can embody the advantages of continuous boosting of a patient’s intrinsic motivation, and reflecting and managing barriers to medication (treatments) on top of psychosocial support and psycho-pharmacological education. This motivational-interviewing-based AT can acknowledge the importance of patients’ involvement in treatment decision and their understanding of both the desirable and negative effects of antipsychotic medication, as well as the possible consequences of adherence or non-adherence to medication, such as unstable mental condition, disturbing and challenging behaviors, and higher risk of relapse [22]. Hence, this insight-inducing and motivation-enhancing approach allows these patients to change their attitude toward medication use, and determine their adherence to medication (treatment) with fully informed, voluntary, and free choices.

### Limitations and future study

A few limitations of this study should be noted. First, while the refusal rate in this study was very low (7 %), over 50 % of the patients in the outpatient clinic were found not to be eligible to participate in this study. In addition, the patients who volunteered to participate were mainly full- or part-time employed (about 60 %), had a relatively shorter duration of illness (64 to 66 % were in their first year of illness), and had satisfactory family support (over 64 % living with family); consequently, they might be highly motivated to participate in AT (indicated by the high attendance rate). The patterns of sociodemographic and illness characteristics, as well as their motivation to change, might also not be representative of Hong Kong and/or other Chinese populations with schizophrenia. Future research with patients with diverse sociodemographic and illness characteristics and/or adherence behaviors, over a longer term follow-up (for example, 12 to 24 months), may allow investigation of the relationships between the perceived benefits, levels of medication adherence (as a mediator), and motivational interviewing techniques, as well as the therapeutic mechanisms of the intervention via individual and/or group interviews and/or structured observations [19].

Second, although we found no difference between the two study groups in terms of the types and dosage of medication taken, we did not record data about side effects, changes in medications and their dosage, and utilization of other mental health services; some of which might have influenced patients’ mental condition and/or insight into their illness and/or treatment. Further research on the confounding effects of these medication- and service-related variables on the effectiveness of AT for people with schizophrenia is recommended.

Third, all of the outcome measures were reported by the participants, except the level of adherence rated by the RA and community psychiatric nurses. Future trials can consider using biological assays such as hair and urine or blood specimens, which are considered more objective and reliable measurements in previous studies [14,25,34], to validate or confirm with the non-invasive, self-reported measures used in this study. Finally, the participants and nurse therapists in this study were not blind to the AT and TAU condition. There was also a lack of control for the frequency and hours of home visits by the community psychiatric nurses in the two study groups, and the working experiences of the nurse therapists in the CPNS. Patients’ preconceived benefits of the AT and the Hawthorne effect due to their awareness of the interventions received might have affected the participants’ (and nurse therapists’) perceived benefits, the participants’ efforts and/or responses and enthusiasm to the interventions, and thus their reported outcomes.

## Conclusions

This controlled trial has been successful in utilizing the strengths of motivational interviewing-based AT for people with schizophrenia spectrum disorders to improve a variety of patient outcomes, including not only patients’ symptom severity and medication adherence, but also their functioning, insight to illness and/or treatment, and number of re-hospitalizations, over a six-month follow-up period. The findings provide evidence on the positive effects of this AT program for schizophrenia sufferers who present with mild to moderate psychotic (both positive and negative) symptoms and poor adherence to medication regimens in psychiatric outpatient clinics. AT, which is a structured and self-empowering model of psychosocial intervention used in conjunction with psycho-pharmacological and other psychiatric treatments, has explicit benefits and applicability to Chinese patients with schizophrenia and other psychotic disorders, and deserves further research into its suitability for a wider implementation in community-based rehabilitation for diverse psychiatric patient groups, and across cultures.

## Abbreviations

ARS: Adherence Rating Scale
AT: Adherence Therapy
CPNS: Community Psychiatric Nursing Services
ITAQ: Insight and Treatment Attitude Questionnaire
PANSS: Positive and Negative Syndrome Scale
SLOF: Specific Levels of Functioning Scale
TAU: Treatment as Usual
